# Cultural inter-population differences do not reflect biological distances: an example of interdisciplinary analysis of populations from Eastern Adriatic coast

**DOI:** 10.3325/cmj.2015.56.230

**Published:** 2015-06

**Authors:** Željana Bašić, Ayano R Fox, Ivana Anterić, Ivan Jerković, Ozren Polašek, Šimun Anđelinović, Mitchell M Holland, Dragan Primorac

**Affiliations:** 1 University Department for Forensic Sciences, University of Split, Split, Croatia; 2 The Pennsylvania State University, Forensic Science Program, University Park, PA, USA; 3 Medical School, University of Split, Split, Croatia; 4 University of Split, Split, Croatia; 5 The Henry C. Lee College of Criminal Justice and Forensic Sciences, University of New Haven, West Haven, CT, USA; 6 Medical School, University of Osijek, Osijek, Croatia

## Abstract

**Aim:**

To compare the population group from the Šopot graveyard with population groups from traditional Croatian medieval graveyards by using anthropological, craniometrics, and mitochondrial (mtDNA) analysis and to examine if the cultural differences between population groups reflect biological differences.

**Methods:**

We determined sex, age at death, pathological, and traumatic changes of skeletal remains from the Šopot graveyard and compared them with a cumulative medieval sample from the same region. We also performed principal component analysis to compare skeletal remains from Šopot with those from Ostrovica and other Central European samples according to 8 cranial measurements. Finally, we compared 46 skeletons from Šopot with medieval (Ostrovica) and contemporary populations using mDNA haplogroup profiling.

**Results:**

The remains from Šopot were similar to the cumulative sample in lifestyle and quality of life markers. Principal component analysis showed that they were closely related to Eastern Adriatic coast sites (including Ostrovica and Šopot) in terms of cranial morphology, indicating similar biological makeup. According to mDNA testing, Šopot population showed no significant differences in the haplogroup prevalence from either medieval or contemporary populations.

**Conclusion:**

This study shows that the Šopot population does not significantly differ from other medieval populations from this area. Besides similar quality of life markers, these populations also had similar biological markers. Substantial archeological differences can therefore be attributed to apparent cultural influences, which in this case do not reflect biological differences.

The process of Christianization in Croatia lasted approximately until the mid-9th century. Duration of this process can be estimated on the basis of changes in burial rituals, since in this period pagan burial rituals were being replaced by Christian rituals (1). Medieval Croatian graveyards after Christianization were typically developed around small churches. The privileged individuals were inhumed near the church and the diseased were inhumed without personal belongings with the exception of clothes and jewelry. Contrary to this, pagan burial rituals included burial with bowls of food and wine, personal belongings, and even animal bones (2,3).

We analyzed the skeletal remains from a portion of the late medieval graveyard in Crkvina, in Šopot near Benkovac (Croatia), dated to the 14th/15th century (3). The site follows the pagan burial pattern, leading archeologists to assume that the remains belonged to a new-comer population, which replaced an autochthonous population wiped out by the white plague (3). The Šopot grave site was excavated for the first time in 1928-1929 by the priest Mate Klarić, who found a pre-romanesque church and graveyard. One of the most important findings from this campaign was an inscription by Duke Branimir, a Croatian medieval ruler, mentioning him as the duke of Croats – *Dux Croatorum* (4). Research on this site continued in 1984, when graves with pagan inhumation ritual were revealed (3,4).

To assess the origin of cultural differences between this graveyard and typical Croatian medieval graveyards after Christianization, we used a multidisciplinary approach, including anthropological, craniometric, nuclear, and mitochondrial DNA (mtDNA) analysis. The first step involved anthropological analysis of the skeletal remains (5) to identify differences between the populations in demographic data, life quality, and trauma. The second step was to compare the Šopot population to other European populations using cranial measurements and determine whether this population group clustered with other Eastern Adriatic coast sites (6). Cranial measurements reflect genetic similarity derived from neutral evolutionary forces, which is why this type of analysis is important in biological distance studies (7), eg, for reconstructing population history, microevolution processes, ancestry, and kinship. Craniometrics is also an important tool in forensic cases for assignment of an unknown skull to a population group (7). Medieval site of Ostrovica was selected for comparison for three reasons: it is considered to be the core of Croatian state in the investigated period and its inhabitants represent the typical Christianized Croatian population; the skeletal remains are well preserved, enabling craniometric measurements and mtDNA analysis; and it is located in the vicinity of Šopot.

The third step involved mtDNA analysis of the skeletal remains from Šopot and their comparison with the results of a previous study on skeletal remains from medieval and contemporary period (8). The previous study included skeletal remains from Ostrovica and is the only published study of mtDNA profiles on medieval skeletal remains from this area (8). In population studies, three classes of DNA markers are typically used: autosomal short tandem repeat (STR) marker, mtDNA sequencing of the control region, and Y-chromosome STR markers. mtDNA sequencing is often most successful for the analysis of ancient skeletal remains or degraded samples since mtDNA is present in each cell in a high copy number. It is also inherited along the maternal line, does not recombine (9), and often exhibits heteroplasmy (the intracellular mixture of variant mtDNA molecules), which can be useful for determination of maternal inheritance inside the graveyard. The main aim of this research was to compare the population groups using anthropological, craniometrics, and mtDNA analysis and to examine if the cultural differences between population groups reflected biological differences.

## Materials and methods

### Setting

Excavations in Šopot revealed 31 graves dated to the 14th/15th century. Around some of the graves and over the majority of the skeletons there was a layer of soot and small fragments of pottery. Also, some of the graves contained animal bones, which is an uncommon finding for a graveyard from this period (3). Excavations in Ostrovica revealed 118 graves, and 128 skeletons were anthropologically analyzed. The graves were dated to the 9th century. Most of the burials were single, which is typical for the early medieval period (10).

### Anthropological analysis

Anthropological and osteometric analysis of skeletal remains from Šopot was performed at the University Department for Forensic Sciences, University of Split in 2011 and 2014. These remains were compared to a cumulative sample of 236 skeletons from the skeletal collection that consists of more than 3000 skeletons. We chose skeletons from 6 medieval Croatian sites: Svećurje-Žestinj (dated to 9/11th century), Rižinice (9/10th century), Bijaći Stombrate (9/10th century), Ostrovica Greblje (9th century), Kamenmost Kaldrma (14/15th century), and Otok Vuletina rupa – Grebčine (17/18th century) (5,10,11), as representatives of typical Croatian graveyards.

Skeletons were washed and bone taphonomy was determined. Sex of the adult skeletons was assessed by examination of the following pelvic features: sciatic notch, ventral arc, subpubic concavity, and ischiopubic ramus (12-16), and by examination of cranial features and long bones (13,14,16,17). When postcranial skeleton was poorly preserved, the analysis of amelogenin gene was performed. The age was estimated using standard anthropological methods (13,14,16,17). In the first step, the age was determined in a wider range by examination of osteodegenerative changes, dental wear, and cranial and palatal sutures, and afterwards more precisely by examination of the pubic symphysis and the auricular surface of the ilium (17). Sex of the children was not determined due to unreliability of sex determination in children (18). The age of subadults was estimated by examination of teeth eruption, diaphysis, epiphysis union, and diaphysis length (17). Skeletons were examined for pathological skeletal changes (especially skeletal evidence of subadult and nutritional stress, and infectious and osteodegenerative diseases), dental pathology, and trauma (19,20).

### Craniometric analysis

Standard osteometric measurements were performed only on completely preserved male crania (21) – 13 from Šopot and 10 from Ostrovica (22). 8 of 24 cranial measurements were chosen for comparison with previously published craniometric data for men, due their availability and the amount of information provided in previous studies (6): maximum cranial length, maximum cranial breadth, minimum frontal breadth, basion-bregma height, bizygomatic breadth, upper facial height, orbital breadth, and orbital height (21). Previously published data originated from 39 central European medieval sites divided in 4 different clusters: Avaroslav sites west of the Danube, Avaroslav sites east of the Danube, Bijelo Brdo sites, and Polish sites (6).

### Nuclear DNA analysis

Analysis of the amelogenin locus (for sex determination) was performed at the Department of Forensic Sciences at the Split University Hospital, using a previously described protocol (11,23,24). For four skeletons from Šopot site, sex was determined using amelogenin gene analysis as sex could not be determined by anthropological methods. Teeth were cleaned and washed with distilled water, dried, cut using a circular saw, and cleaned with 5% commercial bleach. The samples were then frozen using liquid nitrogen and ground into powder. One gram of the powder was used for DNA extraction, with the powder incubated in 0.5 M EDTA for 5 days. DNA was further extracted using extraction buffer (5 mL 100 mM Tris-Cl pH = 8; 5 mL 1 M NaCl; 25 mL 100 mM EDTA pH = 8; 2.5 mL 10% SDS, and dd H20 50 mL), proteinase K, and incubated at 56°C. DNA purification included phenol/chloroform/isoamyl alcohol (25:24:1) and butanol extraction, followed by microfiltration in an Millipore Amicon^®^ (Billerica, MA, USA) filtration device and washing with ddH_2_O. Amplification was performed using the AmpFlSTR® MiniFiler PCR Amplification Kit (Applied Biosystems, Foster City, CA, USA) and the polymerase chain reaction (PCR) was performed in the GeneAmp PCR System 9700 (Applied Biosystems). PCR products were typed on an ABI Prism 310 Genetic Analyzer (Applied Biosystems).

### mtDNA analysis

mtDNA analysis was conducted at Penn State University, Eberly College of Science, Forensic Science Program, USA. It was performed on the control region for 46 preserved skeletal samples from Šopot, and the resulting profiles were compared with previously published results for Croatian medieval and contemporary populations (8) to assess population differences. Skeletal samples were extracted according to the Armed Forces DNA Identification Laboratory bone extraction protocol and required approximately 200 mg of bone powder (25). The samples were incubated overnight with demineralization extraction buffer (0.5 M EDTA, 1% N-lauroylsarcosine, and 0.67 mg/mL proteinase K). Phenol/chloroform/isoamyl alcohol was used to remove protein impurities, and the DNA underwent a final purification and concentration step through an Ultra-4 microfiltration unit with a 30 000 MW cut-off (Millipore Amicon^®^), including washes with ddH_2_O.

Following DNA extraction and purification, the hypervariable regions of the mitochondrial genome were amplified. Due to the antiquity and condition (taphonomy) of the bones, the main component of the extracts was low-quality DNA. For this reason, the hypervariable regions were amplified in two overlapping pieces (26) using the following primer pairs:

F15997 5′-CAC CAT TAG CAC CCA AAG CT-3′

F16159 5′-TAC TTG ACC ACC TGT AGT AC-3′

F29 5′-GGT CTA TCA CCC TAT TAA CCA C -3′

F172 5′-ATT ATT TAT CGC ACC TAC GT-3′

R16236 5′-CTT TGG AGT TGC AGT TGA TG-3′

R16401 5′-TGA TTT CAC GGA GGA TGG TG-3′

R285 5′-GGG GTT TGG TGG AAA TTT TTT G -3′

R408 5′-CTG TTA AAA GTG CAT ACC GCC A-3′

Primer Set I: F15997 / R16236

Primer Set II: F16159 / R16401

Primer Set III: F29 / R285

Primer Set IV: F172 / R408

Following amplification, the products were analyzed on a 2% agarose gel to detect the presence and approximate quantity of the PCR products, as well as the quality of the positive and negative controls. The amplified products were purified by several washes with ddH_2_O through Ultra-4 microfiltration devices. Finally, the products were subjected to routine Sanger-based DNA sequencing performed in the Genomics Core Facility of the Huck Institutes of the Life Sciences at Penn State. The results were compared to previously published data for 100 samples from Ostrovica and 250 profiles from a contemporary Croatian population (8).

### Statistical analysis

The data were analyzed using the statistical package SPSS (version 17, SPSS Inc., Chicago, IL, USA), and the significance level was set at *P* < 0.05. Anthropological data were compared using χ˛ and *t* tests. Craniometric data were compared using principal component analysis (PCA), which is a multivariate technique that transforms a set of correlated variables into a set of uncorrelated variables. In this way, the number of variables in a data set was reduced by finding the linear combinations of those variables that explain the most variability in the sample. mtDNA data were compared using a *t* test (numerical data) and Fisher exact test due to the small sample sizes (categorical data).

## Results

### Anthropological analysis

We compared data on average age at death, diseases, and trauma from 47 individuals (9 female, 23 male, and 15 children) from Šopot and 236 individuals from the cumulative sample (50 female, 92 male, and 94 children) (5). Significant differences were found only in average age at death for men (*P* = 0.003) and the incidence of rickets, which was found in only one skeleton from Šopot (*P* = 0.037) (Table 1).

**Table 1:** Anthropological comparison between Šopot and a cumulative sample of typical Croatian medieval graveyards

	Šopot	Cumulative sample	*P*	χ˛
Average age at death in years (number of cases)*				
male adults	32.4 (23)	40.8 (92)	0.003	-
female adults	28.9 (9)	37.2 (50)	0.337	-
Periostitis (number of cases/number of persons, %)				
children	8/15 (53.3)	69/94 (73.4)	0.491	0.47
adults	6/32 (18.8)	10/142 (7.0)	0.067	4.01
Metabolic diseases				
*Cribra orbitalia* (number of cases/number of persons with preserved orbits, %)				
children	3/7 (42.8)	14/22 (63.6)	0.606	0.265
adults	7/16 (43.75)	18/64 (28.1)	0.399	0.713
Ricketts				
children	0/15 (0)	0/94 (0)	-	-
adults	1/32 (3.13)	0/142 (0)	0.037	4.328
Congenital diseases (*spina bifida*; number of cases/number of persons with preserved sacri, %)				
adults	2/9 (22.2)	2/42 (4.76)	0.119	2.426
Joint diseases				
Osteoartritis (number of cases/number of persons, %)				
adults	15/32 (46.9)	61/92 (66.3)	0.326	0.966
Schmorl’s nodes				
adults	1/9 (11.11)	249/679 (36.67)	0.231	1.433
Dental caries (number of cases/number of persons, %)				
adults	13/21 (61.9)	39/105 (37.1)	0.198	1.654
Trauma (number of cases/number of persons, %)				
children	0/15 (0)	1/94 (1.1)	0.148	2.091
male adults	5/23 (21.7)	18/92 (19.6)	0.850	0.036
female adults	1/9 (11.1)	1/50 (2.0)	0.192	1.704

*t test.

### Principal component analysis of osteometric data

**Figure 1:**
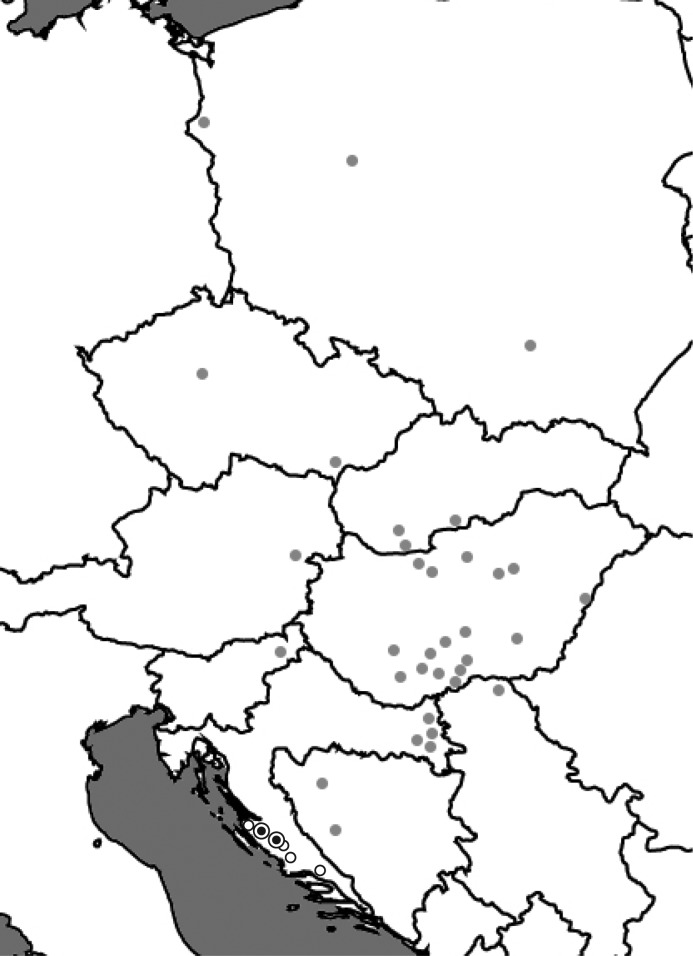
Archaeological sites included in craniometric analysis. Gray circles show previously analyzed continental sites, white circles show previously analyzed Eastern Adriatic coast sites (6), and circled dots present newly added Eastern Adriatic coast sites.

**Figure 2:**
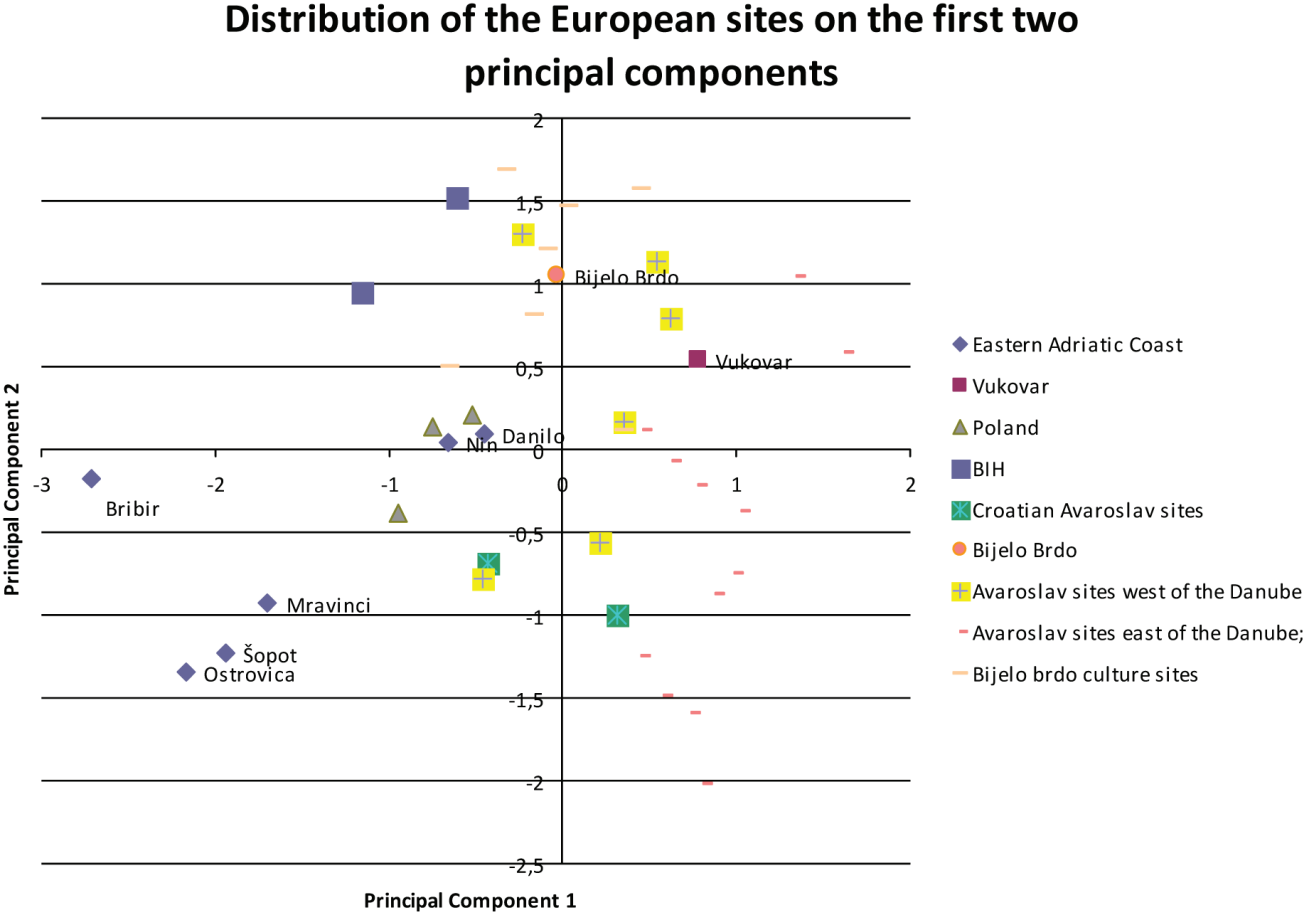
Distribution of the European sites on the first two principal components of cranial measurements.

Cranial measurement data for 13 Šopot and 10 Ostrovica skeletons, as well as previously published craniometric data for medieval central European populations (6) (Figure 1) were compared using PCA (Figure 2). The two first principal components explained 51.35% of the variability in the sample sets. In the first principal component, weight was given to breadth measurements (maximum cranial breadth and bizygomatic breadth). In the second component, almost similar weight was given to basion-bregma height, minimum frontal breadth, orbital breadth, and maximum cranial length. As this component covers all three dimensions (width, height, and length), it is associated with the general size of the cranium. All major variables had positive coefficient signs, which means that the breadth of the cranium increased from left to right on the x-axis, and general cranial size increased from bottom to top on the y-axis (Table 2). Šopot and Ostrovica sites were positioned in proximity to one another and clustered together with other Eastern Adriatic coast sites (Mravinci and Bribir), as well as with Polish sites. These sites were visibly separated from other sites: Avaroslav groups, Bijelo Brdo culture, Bosnia and Herzegovina sites, as well as Croatian continental sites.

**Table 2:** The factor loadings of the craniometric variables in principal component analysis

Measurement	Component	
	1	2
Maximum cranial length	-0.351	0.611
Maximum cranial breadth	0.809	0.044
Minimum frontal breadth	-0.147	0.680
Basion-bregma height	-0.433	0.685
Bizygomatic breadth	0.819	0.132
Upper facial height	0.540	0.223
Orbital breadth	0.346	0.641
Orbital height	0.391	0.320

### Nuclear DNA analysis

Sex was determined by amelogenin analysis for 4 Šopot skeletons. Partial profiles were obtained for 3 samples and a complete profile for one sample. Amelogenin profiles were successfully determined in all 4 cases.

### mtDNA analysis

mtDNA analysis was performed on the control region for 46 individuals. 19 samples yielded complete sequences and 6 samples yielded partial sequences. Of the remaining samples, 13 contained no detectable DNA, 2 showed signs of contamination, and 6 contained inhibition that prevented successful amplification. A total of 13 of the complete mtDNA profiles were sufficient for detailed haplogroup assignment; the remaining 6 profiles mapped to multiple haplogroups. Mitochondrial DNA profiles showed that Šopot population was similar to Croatian medieval and modern populations (8) (Table 3).

**Table 3:** Comparison of the Šopot mitochondrial profiles with previously published results

Haplogroup N (%)	This study (Šopot)	Croatian medieval (7)	*P* (Fisher test)	Croatian modern (7)	*P* (Fisher test)
H	5 (38.46)	62 (62)	0.094	136 (54.4)	0.201
J	3 (23.08)	15 (15)	0.342	31 (12.4)	0.228
U	1 (7.69)	13 (13)	0.450	28 (11.2)	0.569
HV	1 (7.69)	4 (4)	0.464	12 (4.8)	0.491
T	2 (15.38)	2 (2)	0.065	17 (6.8)	0.240
K	0 (0)	3 (3)	0.691	9 (3.6)	0.629
V	0 (0)	1 (1)	0.885	12 (4.8)	0.537
I	0 (0)	0 (0)	NA	5 (2.0)	0.775
N	1 (7.69)	0 (0)	0.115	0 (0)	0.049

## Discussion

To date, there has been no systematic, comparative analysis of anthropological, craniometrics, and mtDNA data from the medieval sites in the Šopot area. Population studies based on only one type of data cannot provide sufficient information on the population dynamics. Therefore, these data require a holistic approach, conjoining multiple aspects that define one population group (genetic heritage, cultural inheritance, geographic/environmental factors etc). Our study suggests that there is no biological difference among the populations of the Eastern Adriatic coast medieval sites.

Overall, these populations showed remarkable similarities. The number of child skeletons was high as children at the time were exposed to numerous diseases, poor living conditions, food deprivation, lower hygiene, inadequate medical care, and population expansion. Both in Šopot and in the cumulative sample we found a high frequency of active periostitis, which is an osteological marker of an unspecific infection (5,10). Also, in both samples (5,10), we found high rates of *cribra orbitilia,* especially in children. C*ribra orbitalia* or orbital roof porosity is a reliable osteological marker of subadult stress, caused by anemia, which is believed to be a consequence of insufficient and/or monotonous diet. An additional confirmation of low nutritional quality is a high frequency of dental caries, indicating a carbohydrate-rich diet, typical for medieval population groups. Another metabolic disease, related to vitamin D deficiency, rickets, was found in one male skeleton from Šopot. Since this is an isolated case it is does not represent a reliable marker of overall population health. We also found cases of *spina bifida* on the sacrum, which is an osteological marker of vitamin B (folic acid) deficiency in pregnancy. The prevalence of *spina bifida* was high in the era before folic acid administration during pregnancy (19). Also, pre-industrial revolution populations often show marks of physical labor, such as muscular stress marks on bones, osteodegenerative changes on joints, and Schmorl’s nodes on the vertebrae. The frequency of Schmorl’s nodes and osteodegenerative changes in the Šopot remains is in accordance with other studies on Croatian medieval populations (27).

Analysis of trauma can indicate the frequency of inter and intra population violence in historical populations. Although the osteological markers of trauma do not represent the total number of injuries, they could be a sensitive marker of changes within a society. There was no significant difference in skeletal trauma between our and previously published results (5,10), suggesting little inter and intra population violence.

Except the age at death for men, anthropological findings in our study suggest similar life conditions in the population groups from Šopot and cumulative sample, including similar diet, health care and hygiene level, physical labor intensity, and inter and intra population violence. As the assessment of life quality and style from skeletal remains is a summation of numerous factors, the difference in only one of these factors is not sufficient to draw conclusions regarding the differences between the population groups.

Anthropological analysis alone cannot provide sufficient evidence about population similarities or differences. However, a valuable contribution in this respect can be given by craniometric and DNA analysis. Genetic drift, gene flow, and natural selection influence the cranial shape and form, and combinations of different types of data (metric, mtDNA, Y chromosome) can be useful for studying population dynamics and historical changes. This was successfully demonstrated by comparison of the osteological and molecular markers in several studies that investigated kinship and human migrations (28-30). When interpreting the relationships within populations, cranial measurement analysis plays an important role and is a valuable aid in studying the morphological variations and relationships among populations (31). Šlaus et al (6) showed using PCA that all of the medieval Adriatic coast sites clustered together and were positioned next to medieval Polish sites (6). Data from their study (6) were included in the PCA in the current study together with data from Šopot and Ostrovica. Our results showed that both sites were closely related to each other and to geographically close sites, Mravinci and Bribir, and clustered with other Eastern Adriatic coast sites. As previously shown (6), all the sites were in vicinity to Polish sites and were separated from the other analyzed sites. PCA showed that all Eastern Adriatic coast sites were closely related in cranial morphology, and thus, most likely had similar biological makeup.

For additional analysis of biological differences and similarities among population groups, we also used mtDNA analysis. Multiple studies have used mtDNA sequences to examine migration patterns of different populations, including Croatians (32,33), Japanese (34), Pacific Islanders (35), Native Americans (36), Australians (37), Siberians (38), and Asian-Pacific populations (39). Similar studies have been conducted using Y-chromosome polymorphisms (40,41). There were no significant differences in mtDNA profiles between Šopot skeletons and skeletons from Ostrovica and Croatian modern skeletons (8). mtDNA analysis additionally confirmed that profiles inherited by the maternal line differed neither between Ostrovica and Šopot site nor between medieval and modern populations, confirming a similar genetic makeup.

One of the limitations of this study was a rather small sample size from the Šopot site, which was a consequence of the small number of skeletal remains and the overall success rate of mtDNA analysis. Unfortunately, meaningful haplogroup information was produced for only 13 Šopot skeletons, which reduced the number of viable comparisons. Interestingly, no maternal relationships were identified between the 25 skeletons that produced mtDNA profile information, although the lack of overall results may have contributed to this finding. For 21 individuals no results were obtained, 6 of which were children, so the age at death may have been a contributing factor. The soil at the site is clay-like type of soil, often rich in metal ions and positive ionic species, which can be deleterious to DNA (42). However we did not perform soil analysis, so it is not clear whether soil type could have contributed to the lack of results.

In conclusion, our anthropological analysis did not confirm the archeological assumption that the Šopot population differed from a typical medieval Croatian population group. PCA analysis showed similarities in cranial shape of all Eastern Adriatic coast populations, including the Šopot population. Craniometrics results were also confirmed by mtDNA analysis, according to which, Šopot population showed no significant differences in the haplogroup prevalence from either medieval or contemporary populations. It is important to stress that the aim of this study was not the comparison and validation of the different methodologies, as the sample size was not large enough for this. Instead, the methods we employed allowed us to confirm or reject our null hypothesis. Taking into consideration all the data obtained by this multidisciplinary research, we advise that historical reconstruction studies distinguish cultural from biological aspects of the population. Although some cultural differences can be found within populations, this does not necessary indicate that they have dissimilar biological structure. In conclusion, the Šopot population has similar biological but different cultural make-up from traditional medieval Croatian population groups.
